# Automatic Deep Learning Semantic Segmentation of Ultrasound Thyroid Cineclips Using Recurrent Fully Convolutional Networks

**DOI:** 10.1109/access.2020.3045906

**Published:** 2020-12-18

**Authors:** JEREMY M. WEBB, DUANE D. MEIXNER, SHAHEEDA A. ADUSEI, ERIC C. POLLEY, MOSTAFA FATEMI, AZRA ALIZAD

**Affiliations:** 1 Department of Radiology, Mayo Clinic College of Medicine and Science, Rochester, MN 55905, USA; 2 Department of Physiology and Biomedical Engineering, Mayo Clinic College of Medicine and Science, Rochester, MN 55905, USA; 3 Division of Biomedical Statistics and Informatics, Mayo Clinic College of Medicine, Rochester, MN 55905, USA

**Keywords:** Deep learning, semantic segmentation, thyroid nodule, thyroid volume, ultrasound, recurrent neural networks

## Abstract

Medical segmentation is an important but challenging task with applications in standardized report generation, remote medicine and reducing medical exam costs by assisting experts. In this paper, we exploit time sequence information using a novel spatio-temporal recurrent deep learning network to automatically segment the thyroid gland in ultrasound cineclips. We train a DeepLabv3+ based convolutional LSTM model in four stages to perform semantic segmentation by exploiting spatial context from ultrasound cineclips. The backbone DeepLabv3+ model is replicated six times and the output layers are replaced with convolutional LSTM layers in an atrous spatial pyramid pooling configuration. Our proposed model achieves mean intersection over union scores of 0.427 for cysts, 0.533 for nodules and 0.739 for thyroid. We demonstrate the potential application of convolutional LSTM models for thyroid ultrasound segmentation.

## I. INTRODUCTION

The incidence of thyroid cancer has been growing across the world for the last 30 years. Incidence rates vary from country to country with an average rate of 9.3 cases per 100,000 in women and 3.1 cases per 100,000 in men [1]. The increase in incidence is attributed to increased access to healthcare [2]. The United States Preventative Services Task Force states the risks of screening asymptomatic adults likely outweighs potential benefits and recommends more conservative strategies including monitoring [1]. Risks associated with thyroidectomy include hypoparathyroidism, infection, and permanent hoarseness or weakness of the voice due to nerve damage [3]. Ultrasonography is the most commonly used diagnostic tool for thyroid cancer as it is inexpensive, non-invasive, portable and widely available [4]. A typical ultrasound thyroid exam involves the creation of a cineclip, a video recording of a full sweep of the ultrasound transducer on each side of the neck viewing the transverse plane of the thyroid. Any lesions detected in the cineclip will be noted, mapped, imaged in different planes and scored. Ultrasonic features of nodules and cysts are quantified to arrive at a numerical estimation of malignancy known as the Thyroid Imaging Reporting and Data system (TI-RADS) [5]. The TI-RADS score helps the physician decide whether to monitor the lesions or to pursue a biopsy. Sonographic features that are suggestive for malignancy include hypoechoic nodules, solid nodules without cystic components, nodules that are taller than wide, irregular margins and presence of calcifications. Sonographic features suggesting a benign pathology include the presence of peripheral vascularity, a round shape, hyper- or iso- echogenicity, spongiform appearance, smooth margins and cystic composition [6]. Estimating scores associated with these features can be subjective and operator dependent. Approximately, 24% of fine needle aspiration (FNA) biopsies of thyroid nodules are indeterminate or non-diagnostic [7]. Following an inconclusive biopsy, a surgical biopsy involving a partial or total thyroidectomy may be recommended. Thus, accurately and consistently measuring the size, volume, and shape of nodules plays a crucial role in optimally recommending treatment.

This paper is organized as follows. Section II introduces several deep learning approaches to 3D datasets. Section III provides an overview of creation of the proposed model; including dataset, model architecture and training details. Section IV discuses model results and finally, Section V provides the conclusion.

## II. BACKGROUND

Video segmentation is a relatively new area of interest in deep learning due to the limitations of high quality labeled video datasets and of memory that compound when moving from static images to video. Early deep learning approaches for handling video data included using top performing 2D models on individual frames. Clocknet proposes a 2D convolutional model adapted for video segmentation by scheduled processing of layers to account for temporal consistency across frames [8]. Models like 3D-Unet and Vnet extend 2D models by replacing 2D convolutional layers with 3D convolutions and depending on the data, treat the time component as a spatial dimension [9]–[15]. Fully 3D models typically must decrease the input size or total number of filters relative to 2D models to conserve memory. The novel MaskTrack model inputs predictions from the previous frame as an input alongside the current frame to incorporate spatio-temporal information [16]. There are also 2D-3D hybrid approaches that use group convolutions to combine multiple frames before feeding the result into a 2D model. From those, the 2D convolutions PedNet [17] approach feeds multiple frames into multiple inputs before merging into a conventional 2D model. Another 2D-3D hybrid is Mnet [18], which is a 2D model with a 3D input operating over adjacent frames. Other hybrid approaches like NetWarp combine optical flow analysis with deep learning. When applied to medical segmentation 3D volumetric approaches have been popular [10]–[15]. There has been development in models implementing recurrent memory units that had initially been used in natural language applications. The STFCN model adapts the recurrent memory units used in natural language applications by manually defining tens of recurrent units, each operating over a small window of backbone model feature maps. More recent recurrent convolutional neural network (CNN), RCNN models take advantage of the development of convolutional long short-term memory (LSTM) layers which replace dozens of LSTM layers with a single LSTM unit applied in a convolutional fashion. FCN-LSTMnet adapts a Unet model by applying two convolutional LSTM to the feature maps of 28 sequential images [19]. The STGRF model uses two groups of backbone models; one group passes information forward over past frames and the second group passes information backwards over future frames before combining intermediate outputs to produce a prediction for the current frame [20]. The BD-LSTM model performs action recognition by examining every sixth frame and using two layers of convolutional LSTM layers to pass information backwards and forwards over multiple frames, and to capture higher level sequence information [21].

## III. METHODS

### A. PATIENT POOL

This prospective study was conducted from September 2015 to September 2017 under an Institutional Review Board approved protocol and was Health Insurance Portability and Accountability Act compliant. A total of 198 cineclips were recorded in 120 patients from one or both sides of the neck during one or more thyroid exams. The imaging protocol consisted of gathering cineclips with a sweep of the thyroid gland in the transverse plane, including some distance beyond the thyroid by a board certified sonographer with more than 30 years of experience scanning thyroids. The cineclips were fully segmented for this study by a team including a trained sonographer. In this study the clinically significant classes, cysts, solid nodules and thyroid, were chosen for segmentation with calcifications considered to be part of the solid nodule class and Hashimoto’s and Grave’s disease not considered to be solid nodules. Patients who were experiencing a nodule recurrence after a partial or total thyroidectomy were removed from the prospective dataset as there were insufficient cases for training. The dataset was divided into independent training, validation and test sets separated by patient such that no patient appears in more than one dataset. Rare pathologies (N<3; metastatic medullary carcinoma, chondrosarcoma, adenomatous nodule) and patients with inconclusive biopsies were first sorted into the training set and the remaining patients were randomly sorted into training, validation and test sets such that the proportion of each known pathology were approximately equal. The pre-training dataset was a superset comprised of the training dataset and cineclips where only the thyroid had been segmented and discarded after pre-training. The test set was hand segmented by an expert sonographer with 30-plus years’ experience. Due to the quantity of data in the test set and the cost of hand segmenting data only every 20th frame from the first appearance of the thyroid until the thyroid vanished from view were segmented. The training set and validation sets consisted of fully segmented cineclips including frames before and after the thyroid entered the viewing plane. The height and width of each cineclip depends on the specific hardware and settings used to acquire the ultrasound data. Cineclips were resized by setting the largest dimension to 256 pixels and the shorter dimension was resized to preserve the original video’s aspect ratio. Empty space was filled with −1 to help the model distinguish between very dark regions at the edges of the frame and the filler.

### B. MODEL ARCHITECTURE

**FIGURE 1.**
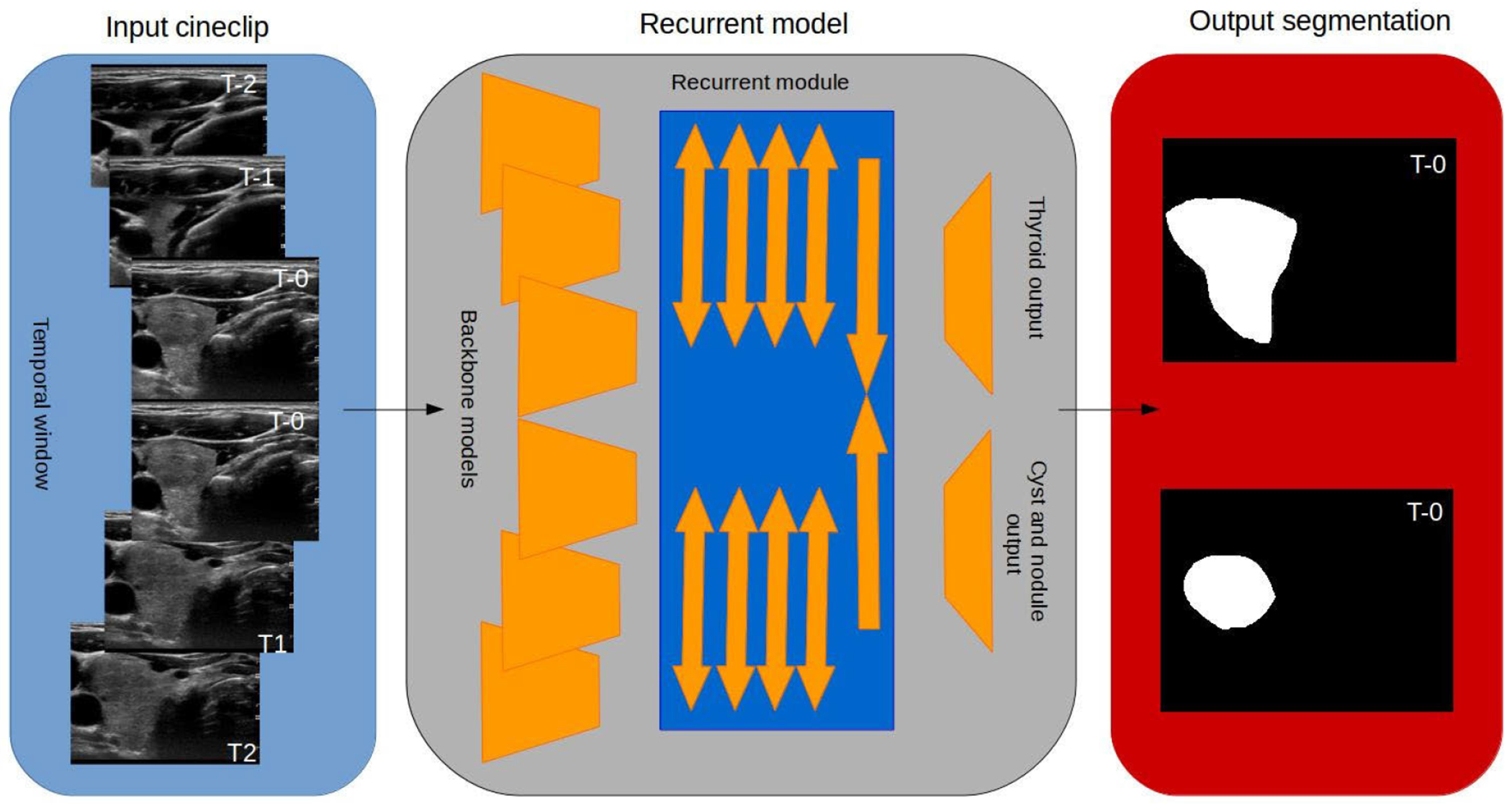
Block diagram of the proposed model. Orange trapezoids represent the backbone DeepLabv3 + models with the output layers removed. The blue rectangle represents the recurrent module with arrows representing convolutional LSTM layers. The inverted orange trapezoids represent the upsampling and output layers.

The backbone model adapted for the recurrent convolutional neural network (RCNN) is the top performing segmentation network DeepLabv3 + [22] using ResNet101 with the ResNet-C input stem replacement [23]. Additional changes to the ResNet101 model included replacing the downsizing strided convolutional operations in the last two blocks with dilated convolutions to expand context without sacrificing spatial resolution or memory; strided by two and four respectively. All ReLu activation functions were replaced with Leaky ReLu activations with an alpha value of 0.10. The model output used transposed convolutions with a stride of 8 × 8 and kernel size of 4 × 4 to return the output size to that of the model input. Improved performance was observed when using ResNet152 and removing the first maxpool operation, however the memory use exceeded resources when creating the final time series model. The model had two outputs; the first output used a sigmoid activation function to segment the thyroid and the second output use a softmax activation to segment the cysts and nodules. Fig. 1 displays a block diagram of the model showing how the backbone model is adapted for time series semantic segmentation. In our previous study [24] it was found that using a dual output model structure improves segmentation performance in medical ultrasound applications. The benefits of using multiple outputs in ultrasound medical segmentation are twofold: multiple outputs simplify post-processing and the nature of medical semantic segmentation supports the framework. Common deep learning applications of semantic segmentation deal with exclusive categories (i.e. person, building, car, etc.) whereas in medical segmentation the classes may be shared such as tissue nodule within thyroid tissue compared to thyroid tissue. It was observed that in some challenging cases where nodules presented ill-defined margins the model would output uncertain predictions at the probable boundaries of the thyroid and nodule with equal weighting between classes rather than a smooth transition from one class prediction to another. Applying a simple thresholding operation resulted in boundary regions with an unrealistic “patchwork” of thyroid and nodule tissue due to slight variations in the prediction. Using a standard single output model formulates the segmentation problem as one in which we must define the outer boundaries of nodules, define the inner boundaries of the thyroid around the nodule and define the outer boundaries of the thyroid. By using a dual output model the problem formulation is simplified by removing the need to define the inner boundaries of the thyroid around lesions.

The proposed algorithm modification produced higher mean results and more certain segmentations, particularly when segmenting lesions with ambiguous margins. The backbone model was adapted for time series segmentation by freezing all layers, removing the upsizing output layers and replicating the model six times. A recurrent module was appended to the new single feature map output of each backbone model. The recurrent module was adapted from the STGRU [20]. The recurrent module was made up of 18 convolutional LSTM layers each with 3 by 3 kernels and 32 filters. Pairs of convolutional LSTM layers form a block; one operating in a forward fashion and one operating backwards to transmit information backwards and forwards. The pairs were then concatenated, normalized, and Leaky ReLu activation was applied. Four stacks of blocks were applied with a dilation rate of 1 × 1, 3 × 3, 5 × 5 and 7 × 7 to both three model clusters. Finally two convolutional LSTMs were applied to the two model clusters to combine the outputs and the final segmentation was obtained through the dual output as previously described.

### C. MODEL TRAINING

Class imbalance is a known issue in deep learning and common in medical applications. As the quantity of data increases the portion of healthy tissue and non-gland background tissue increases greatly relative to the quantity of unhealthy tissue. Unaddressed, this leads to the model heavily prioritizing the common classes over the more important and rarer classes. The current dataset has nodules in approximately 35% of frames and approximately 2% of the dataset by pixel count. Three methods were considered to combat class imbalance in this study: data sampling, algorithm modification, and cost-sensitive learning.

Subsampling chooses all examples of the minority class and samples from the majority class at a reduced rate and potentially risks discarding useful data. Oversampling increases the quantity of data by repeating examples of the minority class, either with or without augmentation and potentially risks overfitting. Hybrid techniques are any combination of over- and sub- sampling. In our tests subsampling was performed by controlling the quantity of nodules in each batch. Oversampling was performed by doubling the quantity of the minority class with horizontal flipping data augmentation. In our tests subsampling the dataset to 60% nodule improved performance over full sampling, but oversampling with data augmentation improved performance across all metrics over subsampling.

Cost sensitive learning modifies the loss function to penalize missed predictions of minority classes greater than majority classes. We experimented with down weighting the background and up weighting classes with the inverse of frame-frequency and inverse of pixel-frequency. Combining the class up weighting and background down weighting seemed to increase instability in training and decreased overall performance. Down weighting the background improved performance, while up weighting classes by the inverse of pixel frequency improved performance with a tendency of over segmentation and decreasing performance in the majority class. A number of new loss functions have been proposed that are designed to address class imbalance. Generalized Dice coefficient loss, sensitivity-specificity loss, Tversky loss, Focal loss, Asymmetric log loss, Combo loss, Lovasz hinge, Boundary F1 loss and mean Hausdorff distance [15], [25]–[30]. The best performance was achieved with a weighted loss function adapted from Matthew’s correlation coefficient (MCC), which rewards true positives and true negatives, and penalizes false positives and false negatives. The advantage of using the MCC loss function is that it provides useful back propagation when there is no true positive present, while other overlap based loss functions return zero whether the model correctly predicts no true positive or predicts a large false positive.

Model training was performed in four stages using the augmentation schemes shown in Table 2. The first stage is pre-training of a single-output single-class version of the model using batches drawn from a larger dataset of healthy and diseased thyroids. The pretraining dataset seen in Table 1 is larger than the final dataset due to the relative ease in segmenting the thyroid compared to nodules and cysts. The second stage introduced the second output to the model, and retrained the model using batches drawn from the training set using data augmentation shown in Table 2, and oversampling frames with the nodule by 66%. That is two batches of data chosen to include a nodule for every three batches of randomly sampled data. The third stage froze the model up to the ASPP module [22] and trained the remaining output layers on full sequences of cineclips from the training set as recommended by [31] to combat class imbalance without bias towards over segmentation. The fourth stage of training modified the pretrained backbone model into the time series model and trained on full sequences of cineclips randomly sampled from the training set.

## IV. RESULTS & DISCUSSIONS

**FIGURE 2.**
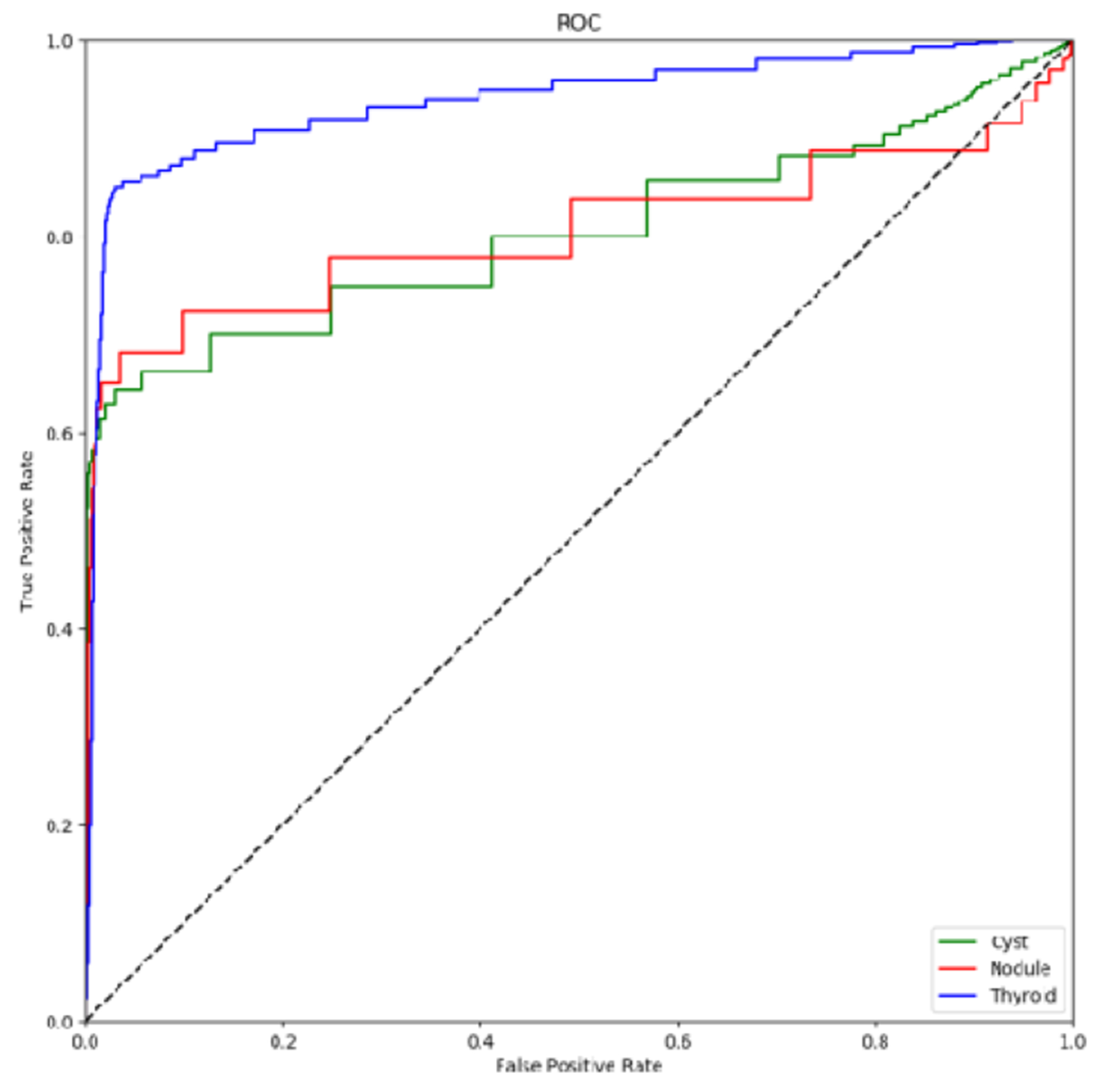
ROC curve for the three segmented features; cyst in green, nodules in red and thyroid in blue.

Fig. 2 shows the ROC curve for the segmented cysts, nodules and thyroids which demonstrates high overall performance across the features of interest in the test set. As in [24], the proposed model results are compared against a conventional seeded segmentation algorithm, the distance regularized level set (DRLS) algorithm presented by Li *et al.* [32]. The parameters were optimized for ultrasound thyroid segmentation using grid search and the results are: time step of 1.0, lambda of 1, alpha of −0.9, epsilon of 2.75, outer loop of 60 iterations and 10 refinement iterations. The segmentation seeds were obtained by dilating the ground truth mask using a 20 pixel disk structure. The high recall and low performance across other metrics suggests strong tendency toward over segmentation. Even when provided guidance with a seed derived from the ground truth mask the algorithm fails to finds boundaries in most cases.

Table 3 shows mean and standard deviation values comparing the results of the proposed model, the MPCNN model and the DRLS algorithm for the five metrics used to evaluate model performance. All metrics are defined in (1–5) in terms of true positive, true negative, false positive and false negative. Intersection over union (IoU) is defined in (1) and is a commonly used segmentation metric that measures the degree of overlap between the prediction and ground truth.

(1) $$\mathrm{IoU}=\frac{\mathrm{TP}}{\left(\mathrm{TP}+\mathrm{FN}+\mathrm{FP}\right)}$$

(2) $$MCC=\frac{TP*TN-FP*FN}{\sqrt{\left(TP+FP\right)\left(TP+FN\right)\left(TN+FP\right)\left(TN+FN\right)}}$$

(3) $$Recall=\frac{TP}{\left(TP+FN\right)}$$

(4) $$Precision=\frac{TP}{\left(TP+FP\right)}$$

(5) $$F2=\frac{5TP}{\left(5TP+4FN+FP\right)}$$

MCC is defined in (2) and is a metric typically used in classification tasks. MCC incorporates true positive, false positive, true negative and false negative to provide valuable information when dealing with sparse segmentations when the presence of positive classes is uncommon such as medical segmentation. Recall is defined in (3) and measures the correctly segmented pixels of a feature compared to all pixels belonging to a feature. Precision is defined in (4) measures the correctly segmented pixels of a feature compared to all pixels predicted to belong to that feature. The F2 measure is a member of a family of F-measures that combine precision and recall with different weights. The F2 measure is defined in (5) and places greater importance on recall than precision which is of interest in medical segmentation as the cost of false negatives is greater than a false positive. Our model is compared to MPCNN, a VGG16 based semantic segmentation model designed from ultrasound medical image segmentation. The proposed model outperforms the MPCNN model across all metrics. The proposed model had an increase in mean MCC metric of 0.31, 0.18 and 0.56 for cysts, nodules and thyroid respectively. Low performance of the MPCNN model in the cyst and nodule metrics were largely due to frames in which small and ambiguous frames when features were entering or leaving the frame. The MPCNN model was trained on ultrasound images provided by clinicians rather than cineclips. The protocol for collecting images is to image the largest cross-section in one or more planes. The effect is that the MPCNN operating on its dataset was partially guided with almost every frames having a nodule or cyst, and presented as clearly as the operator could manage. In contrast, cineclips present less ideal scenarios.

In Table 4, model results are compared against the echogenicity of the nodule for each patient. Hypoechoic denotes that the nodule is darker than the surrounding tissue and is frequently observed, hyperechoic denotes that the nodule is brighter than the surrounding tissue and isoechoic denotes that the nodule has the same gray level as the surrounding tissue. The metrics compared against hypoechoic and isoechoic nodules indicates the model’s ability to correctly distinguish between nodules and cysts which may possess clutter but otherwise appear hypoechoic and isoechoic. The metrics compared against hyperechoic nodules indicates the model’s ability to correctly identify nodules and distinguish the boundaries from the thyroid. Table 4 shows an unexpected inversion as cyst segmentation performs best when the nodule has lower contrast. Fig. 4b shows that the segmentation of nodules performs best when the nodules are hypoechoic and having less contrast with the surrounding thyroid tissue. Table 4 shows the segmentation results of the overall thyroid and is relatively indifferent to the condition of the containing nodule. This is a benefit of the model’s dual output as there is no tradeoff between the thyroid and other classes. The proposed model outperforms the MPCNN model due to advancements in the base backbone model, the recurrent module, training method and loss function. The new loss function is a class balanced MCC modified as a loss function. In testing it was found that down weighting the background and thyroid classes, which greatly out numbers the nodule and cysts classes, improved performance in the minority classes without sacrificing performance in the majority classes. The MCC loss provides a useful gradient update even when there is no true positive whereas traditional overlap based loss functions return zero when there is no true positive whether the model over segments or correctly outputs no segmentation.

The new backbone model, a modified ResNet 101 model with Atrous Spatial Pooling module, offers greater performance than the VGG16 based model with lower memory footprint. Six pretrained copies of the backbone model are modified by replacing the output layers with a series of convolutional LSTM layers that allow the model to share features collected from five consecutive frames before outputting the segmentation map for the current frame. The approach outperforms both 2D and 3D versions of the model. The LSTM model is of additional benefit in ultrasound as the focal depth of the probe means that each frame represents a non-zero slice of tissue. When viewed in a cineclip a sweep across tissue will often show healthy thyroid tissue darken from the presence of a nodule or cyst adjacent to the viewing location. The transitional phases as features leave and enter the viewing plane cause confusion in the previous model. Blood vessels branching from the main arteries and running through the thyroid can often be difficult to distinguish from small cysts and can require time series information from multiple frames to see if the feature persists. As with the old model, the new model struggles with hyperechoic nodules and nodules that replace the entirety of the thyroid. In these cases the model detects the presence of a nodule, but under segments the area.

In Table 5 the results are compared against the malignancy of the nodule. Malignant nodules are more likely to have indistinct boundaries, projections and more complex features presenting more difficult objects to segment and critically important features. Table 5 shows approximately equivalent results in the nodule for both benign and malignant nodules with a slight trend towards higher maximum performance in benign nodules. Table 5 shows approximately equal results in the thyroid regardless of the malignancy in the nodule.

In Table 6 metrics for the nodule and thyroid are shown compared against the margins of the nodules; smooth, ill-defined or lobulated. The margin features are used when estimating the malignancy of nodules, with smooth margins suggesting benign, and ill-defined and lobulated margins suggesting malignancy. Given the distribution of the test set there was one patient with a nodule having ill-defined margins. Table 6 shows that there is little change in the performance in segmentation of the cyst class with regards to the margins of the nodule as expected. Table 6 shows roughly equal performance in the nodule class with regards to the margins of the nodule with a clear improvement in the precision of nodules with smooth margins.

**FIGURE 3.**
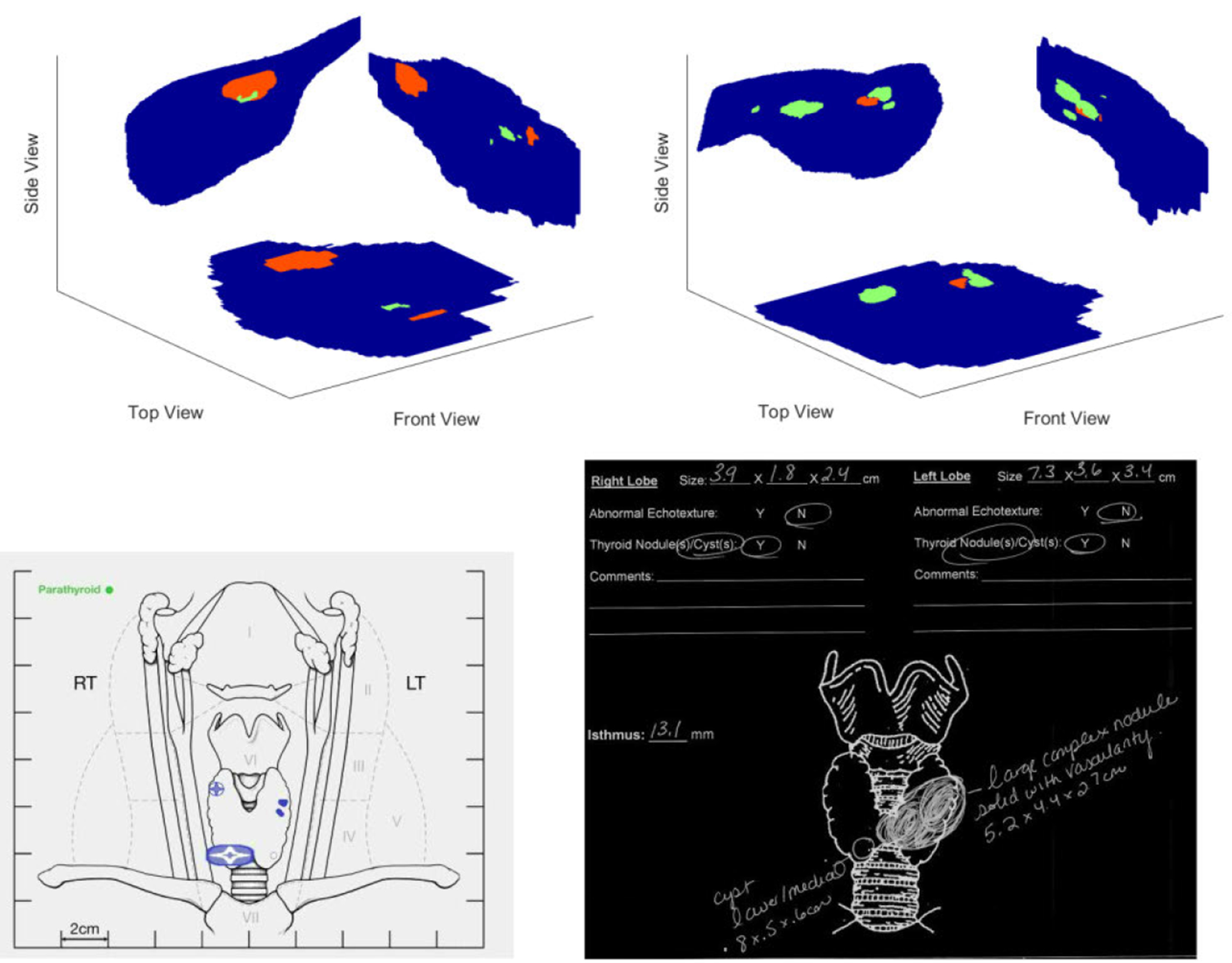
Examples of Comparison of automatically generated thyroid maps in multiple planes with thyroid maps generated by sonographers. In the generated maps blue represents the thyroid gland, red represents solid nodules and green represents cysts.

Fig. 3 displays one of the potential applications of the ultrasound thyroid semantic segmentation model to help automate mapping of the thyroid. Once segmented, the resulting 3D thyroid volume can be viewed along any plane and orientation. Here, three standard engineering planes were created by summing each class along the X, Y and Z axes. The size and volume of each class can be calculated by integrating the area of each slice over the length of the thyroid found by measuring the length of the thyroid in a longitudinal plane and assuming a constant uniform velocity. Currently the dimensions of nodules and large cysts are measured in two perpendicular planes and the volume of the thyroid is estimated using an ellipsoid approximation. Comparing the performance of the ellipsoid approximation with an integral calculation results in a mean percent difference of −13.55%. This result correlates well to a study by Vurdem *et al.* [33] comparing 2D ultrasound thyroid volume estimation with the volume as measured by post-thyroidectomy which found ultrasound thyroid volume estimation had a systematic underestimation of the thyroid by 10.62%

## V. CONCLUSION

In this paper, we present a novel recurrent semantic segmentation network suitable for automatic segmentation of thyroid ultrasound cineclips. Our proposed method incorporates the top performing DeepLabv3 + model, a novel recurrent module, a novel model for segmentation MCC loss function and a training procedure. In contrast to previous papers our proposed method takes advantage of the format of the typical thyroid ultrasound exam. Segmentation performance of the thyroid feature using our proposed model is very high, but the performance in cysts and nodules are not yet acceptable to be used as an assistance tool. We expect performance to increase with larger datasets. Kohl *et al.* has proposed techniques specifically designed for ambiguous segmentation as encountered in some nodules with ill-defined margins [34]. Karimi *et al.* and Abraham *et al.* propose modifications to standard segmentation loss functions to improve performance over the unmodified loss functions [29], [35]. Such modifications could potentially be applied to our class balanced MCC loss to further improve performance on cysts and nodules. Regularization had not been applied to our model, but has been shown to improve model performance in certain applications. A more accurate segmentation model could be used in clinical work, either directly implemented into commercial ultrasound systems or implemented as a separate post-processing step. A live implementation could assist with remote medical clinics where expertise may be limited. Given the ambiguous margins of some nodules a consistent, unified segmentation tool may help improve consistency of the TI-RAD system used to recommend further application.

## Figures and Tables

**TABLE 1.** Distribution of number of patients, cineclips and frames in each dataset.

Dataset	Patient	Cine clips	Frames
Pre-Training	87	146	40,168
Training	64	90	23,899
Validation	18	24	6,063
Test	15	28	342

**TABLE 2.** Augmentation Schemes and Settings used during Training.

Stage	Scheme	Frequency	Scale
Stage 1	Horizontal Flipping	50%	-
	Rotation	25%	15
	Translation	25%	10%
	Color shift	10%	5%
	Crop	100%	300 pixels
Stage 2	Horizontal Flipping	50%	-
	Translation	25%	10%
	Color shift	10%	5%
	Crop	100%	256 pixels
	Oversampling	66%	Nodule
Stage 3	Horizontal Flipping	50%	-
	Rotation	50%	5
	Translation	25%	10%
	Color shift	10%	5%
	Crop	100%	256 pixels
Stage 4	Horizontal Flipping	50%	-
	Reverse sequence	50%	-

**TABLE 3.** Comparison of results between proposed model, mpcnn model and DRLS algorithm for all metrics over each reported feature.

Feature	IoU	MCC	Recall	Precision	F2	Method
Cyst Mean	**0.417**	**0.517**	**0.604**	**0.823**	**0.570**	Proposed Model
Cyst Std.	0.286	0.302	0.378	0.220	0.336	
Nodule Mean	**0.533**	**0.612**	0.653	**0.845**	**0.663**	
Nodule Std.	0.269	0.277	0.321	0.236	0.290	
Thyroid Mean	**0.739**	**0.813**	0.869	**0.843**	**0.846**	
Thyroid Std.	0.214	0.205	0.211	0.161	0.214	
Cyst Mean	0.029	0.070	0.262	0.040	0.355	MPCCN model
Cyst Std.	0.055	0.121	0.360	0.088	0.355	
Nodule Mean	0.069	−0.028	**0.996**	0.069	0.230	
Nodule Std.	0.073	0.028	0.005	0.073	0.182	
Thyroid Mean	0.142	−0.023	**0.999**	0.142	0.422	
Thyroid Std.	0.074	0.019	0.002	0.074	0.162	
Cyst Mean	0.222	0.357	0.999	0.222	0.438	DRLS algorithm
Cyst Std.	0.194	0.111	0.000	0.194	0.188	
Nodule Mean	0.396	0.538	0.999	0.405	0.683	
Nodule Std.	0.121	0.099	0.000	0.113	0.150	
Thyroid Mean	0.451	0.595	0.999	0.450	0.787	
Thyroid Std.	0.112	0.081	0.000	0.112	0.112	

**TABLE 4.** Results for each reported feature by echogenicity as determined by a radiologist.

Feature	IoU	MCC	Recall	Precision	F2	Echogenicity
Cyst Mean	-	-	-	-	-	Hyperechoic
Cyst Std.	-	-	-	-	-	
Nodule Mean	**0.667**	**0.758**	**0.800**	0.853	**0.780**	
Nodule Std.	0.243	0.251	0.272	0.168	0.258	
Thyroid Mean	**0.773**	**0.843**	**0.913**	0.814	**0.922**	
Thyroid Std.	0.176	0.175	0.184	0.191	0.046	

Cyst Mean	**0.441**	**0.533**	**0.616**	0.808	**0.583**	Hypoechoic
Cyst Std.	0.261	0.293	0.373	0.226	0.323	
Nodule Mean	0.524	0.470	0.662	0.844	0.672	
Nodule Std.	0.279	0.339	0.311	0.220	0.276	
Thyroid Mean	0.746	0.820	0.873	**0.847**	0.848	
Thyroid Std.	0.204	0.191	0.190	0.163	0.203	

Cyst Mean	0.389	0.469	0.441	**0.941**	0.394	Isoechoic
Cyst Std.	0.349	0.323	0.409	0.070	0.353	
Nodule Mean	0.433	0.386	0.515	**0.894**	0.469	
Nodule Std.	0.221	0.307	0.297	0.114	0.293	
Thyroid Mean	0.707	0.778	0.835	0.846	0.807	
Thyroid Std.	0.263	0.260	0.274	0.152	0.282	

**TABLE 5.** Comparison of results separated by malignancy of the nodules.

Feature	IoU	MCC	Recall	Precision	F2	Malignancy
Nodule Mean	**0.533**	**0.612**	0.653	**0.845**	**0.663**	Benign
Nodule Std.	0.269	0.277	0.321	0.236	0.290	
Thyroid Mean	**0.739**	**0.813**	0.869	**0.843**	**0.846**	
Thyroid Std.	0.214	0.205	0.211	0.161	0.214	

Nodule Mean	0.069	−0.028	**0.996**	0.069	0.230	Malignant
Nodule Std.	0.073	0.028	0.005	0.073	0.182	
Thyroid Mean	0.142	−0.023	**0.999**	0.142	0.422	
Thyroid Std.	0.074	0.019	0.002	0.074	0.162	

**TABLE 6.** Comparison of results separated by nodule margins as determined a radiologist.

Feature	IoU	MCC	Recall	Precision	F2	Margins
Cyst Mean	-	-	-	-	-	Ill-Defined
Cyst Std.	-	-	-	-	-	
Nodule Mean	**0.667**	**0.758**	**0.800**	0.853	**0.780**	
Nodule Std.	0.243	0.251	0.272	0.168	0.258	
Thyroid Mean	**0.773**	**0.843**	**0.913**	0.814	**0.922**	
Thyroid Std.	0.176	0.175	0.184	0.191	0.046	

Cyst Mean	**0.441**	**0.533**	**0.616**	0.808	**0.583**	Smooth
Cyst Std.	0.261	0.293	0.373	0.226	0.323	
Nodule Mean	0.524	0.470	0.662	0.844	0.672	
Nodule Std.	0.279	0.339	0.311	0.220	0.276	
Thyroid Mean	0.746	0.820	0.873	**0.847**	0.848	
Thyroid Std.	0.204	0.191	0.190	0.163	0.203	

Cyst Mean	0.389	0.469	0.441	**0.941**	0.394	Lobulated
Cyst Std.	0.349	0.323	0.409	0.070	0.353	
Nodule Mean	0.433	0.386	0.515	**0.894**	0.469	
Nodule Std.	0.221	0.307	0.297	0.114	0.293	
Thyroid Mean	0.707	0.778	0.835	0.846	0.807	
Thyroid Std.	0.263	0.260	0.274	0.152	0.282	

**TABLE 7.** Stage 3 model parameters.

	C32 LR BN
	C32 LR BN
	C64 LR BN
Skip1	Maxpool
Skip2	CB [64 64 256]
	2x IB [64 64 256]
Skip3	CB [128 128 256] S2
	4x IB [128 128 512]
Skip4	CB [256 256 1024] D2
	23x IB [256 256 1024] D2
	CB [512 512 2048] D4
	2x IB [512 512 2048] D4
	ASPP
Skip1 C128 F1	C128 F1
	Concat BN LR
Skip2 C128 F1	C128 F1
	Concat BN LR
Skip3 C128 F1	C128 F1
	Concat BN LR
Skip4 C128 F1	C128 F1
	Concat BN LR

**TABLE 8.** Stage 1 and Stage 2 model parameters.

Stage 1 Model	Stage 2 Model	

Stage 3	Stage 3	
DC1 F8 S4	DC1 F8 S4	DC3 F8 S4
C1 F1	C1 F1	C3 F1
Sigmoid	Sigmoid	Softmax

**TABLE 9.** Recurrent module parameters.

Forward Group	Backward Group

3x Stage3 model	3x Stage3 model
CL32 D1 Forward	CL32 D1 Forward
CL32 D1 Backward	CL32 D1 Backward
Concat BN LR	Concat BN LR
CL32 D3 Forward	CL32 D3 Forward
CL32 D3 Backward	CL32 D3 Backward
Concat BN LR	Concat BN LR
CL32 D5 Forward	CL32 D5 Forward
CL32 D5 Backward	CL32 D5 Backward
Concat BN LR	Concat BN LR
CL32 D7 Forward	CL32 D7 Forward
CL32 D7 Backward	CL32 D7 Backward
Concat BN LR	Concat BN LR
CL32 D1 Forward	CL32 D1 Backward

Concat BN LR	
DC1 F4 S8	DC3 F4 S8
C1 F1	C3 F1
Sigmoid	Softmax

**TABLE 10.** Identity and convolutional block parameters.

IB [X Y Z]	CB [X Y Z]	
CX F1	CX F1	CZ F1
BN LR	BN LR	BN
CY	CY	
BN LR	BN LR	
CZ	CZ	
BN LR	BN	
Add input	Add	
LR	LR	

## APPENDIX

Parameters for the proposed model are provided in tables 7–10 below. The stage 3 model is defined in table 7. Stage 1, stage 2 and recurrent models are defined from the stage 3 model in tables 8 and 9. The identity and convolutional blocks are defined in table 10. Let CX denote 2D convolutional layers with X number of filters, DCx denote 2D deconvolutional layers with X number of filters, BN denote batch normalization, concat denote concatenation layer, LR denote Leaky ReLU activation units with an alpha value of 0.10. Let CB [X Y Z] denote a ResNet convolutional block with X, Y and Z number of filters. Let XxIB [W Y Z] denote X stacks of ResNet identity blocks with W, Y and Z number of filters. Let ASPP denote an atrous spatial pyramid pooling module. Let F, S and D denote filter kernel size, strides, and dilation rate. Unless specified otherwise all filters are 3 by 3, a stride of 1 and dilation rate of 1.
